# Tracing the introduction history of the tulip that went wild (*Tulipa sylvestris*) in sixteenth-century Europe

**DOI:** 10.1038/s41598-022-13378-9

**Published:** 2022-06-13

**Authors:** Anastasia Stefanaki, Tilmann Walter, Tinde van Andel

**Affiliations:** 1 Biosystematics Group, Wageningen University, Droevendaalsesteeg 1, 6708 PB Wageningen, The Netherlands; 2 Naturalis Biodiversity Center, PO Box 9517, 2300 RA Leiden, The Netherlands; 3 Institute of the History of Medicine, Julius-Maximilians-Universität Würzburg, Oberer Neubergweg 10a, 97074 Würzburg, Germany; 4 Institute of Biology, Clusius Chair of History of Botany and Gardens, Leiden University, Sylviusweg 72, 2333 BE Leiden, The Netherlands

**Keywords:** Plant sciences, Plant domestication

## Abstract

*Tulipa sylvestris*, commonly called the “wild tulip”, was introduced from the Mediterranean to northern Europe in the sixteenth century and became widely naturalized. Research has focused on tulips that came from the Ottoman Empire, but the introduction path of this native European, early ornamental tulip is unclear, and so is its taxonomic status: three subspecies are provisionally accepted, sometimes treated as species. Here we elucidate the history of introduction of *T. sylvestris* and discuss its taxonomy based on our historical findings. The first bulbs came from Bologna (northern Italy) and Montpellier (southern France) in the 1550–1570 s. Several renowned botanists were involved in their introduction, namely Gessner, Wieland, Aldrovandi, De Lobel, Clusius, and Dodoens. There were various introduction routes, including one from Spain which was apparently unsuccessful. The strong sixteenth-century Flemish botanical network facilitated the introduction and naturalization of *T. sylvestris* across Europe. Based on the latest tulip taxonomy, the diploid subspecies *australis* is native in the Mediterranean, and the tetraploid *sylvestris* is naturalized over Europe, but our historical findings show that both *sylvestris* and *australis* were introduced to northern Europe. This underlines the need to reconsider the taxonomic status of *T. sylvestris*, highlighting the importance of botanical history in understanding the complex taxonomy of naturalized cultivated plants.

## Introduction

Ornamental tulips were introduced to Europe from the Ottoman Empire in the sixteenth century. The tulip tradition was probably brought with the Seljuks on their migration route from Central Asia to Anatolia^1^, and gained increasing popularity in Ottoman gardening and art^2^. Throughout the sixteenth century, several kinds of tulips were brought, sometimes in large amounts, from the empire provinces and abroad^1,3^. For the Ottomans, tulips had a symbolic religious meaning, and became a main ornamental motif in their culture^1^. The tulip trend that spread in the sixteenth-century Ottoman world was soon to be expanded westwards to Europe. The Flemish Ogier Ghiselin de Busbecq (1522–1592), ambassador of the Habsburg emperor in the Ottoman court in Istanbul in 1554–1562, was long acknowledged as the first to bring tulips to Europe. Presumably in 1554, on his way from Edirne to Istanbul, De Busbecq observed a flower unknown to him called “tulipan” by the Turks^4,5^. This event, however, described in his famous *Turkish letters*^4^, might have taken place years later, as the letters were actually written between 1581 and 1589^6^. The French diplomat and naturalist Pierre Belon (1517–1564) may actually have been the first to bring oriental tulips to Europe^1^. In 1553, he wrote about the red lilies (“lils rouges”) that every Turk had in his garden^7^. Belon had a garden himself, with exotic plants which he brought from his travels in the Orient during the 1540 s^1,8^ and distributed in his network.

Once in Europe, tulips soon attracted scholarly attention. In 1559, the famous Swiss naturalist Conrad Gessner (1516–1565) observed a single red tulip that grew in the garden of city councilor Johann Heinrich Herwart in Augsburg^9^, a rich merchant city in Southern Germany. Two years later, he published the first description of a tulip accompanied by a woodcut^10^. The latter was not made after Herwart’s tulip as previously thought, but apparently after an illustration that Gessner had received from one of his correspondents in Augsburg. This illustration was drawn after a tulip in the garden of the rich Fugger family^9^, acquired via the Fuggers’ close social connections with the Habsburg court. Gessner’s tulip was probably *Tulipa suaveolens* Roth.*,* the presumed wild ancestor of the “garden tulip”, i.e., *T. gesneriana* L., a complex garden hybrid, from which modern tulip cultivars have derived^11^. The prominent Flemish botanist Carolus Clusius (1526–1609) published the first tulip monograph in 1576 and amended it twice over the next three decades^12–14^. Clusius vigorously received and distributed tulips within his vast network of correspondents in Europe^15^. In 1593 he moved, together with his tulips, to Leiden, the Netherlands, to take over the city’s university garden, presently the Hortus Botanicus. Clusius’ bulbs were repeatedly stolen despite his efforts to prevent theft and became a main material source for the later Dutch bulb trade^16,17^. By the end of the sixteenth century, a plethora of tulip cultivars, with flowers of variable colors and shapes, had made it to the gardens of royalties and nobles, collectors, scholars, amateurs and professional bulb growers. In the first decades of the seventeenth century, the demand for tulips got absurd dimensions reaching a peak during the so-called Tulipomania, when a handful of bulbs would be sold for prodigious prices^17,18^. The tulip fever ceased in 1637, but it had already laid the foundations of modern international flower trade^18^.

During the same period that ornamental tulips reached Europe from the Ottoman empire, *Tulipa sylvestris* L., a small yellow tulip known then as “*Narcissus luteus*” (yellow daffodil) was also introduced to northern Europe, but following a different path. Unlike the rest of the tulips that came from the east, *T. sylvestris* came from the south, the Mediterranean region^19,20^. It had neither the long pointy tepals that were favored by the Ottomans^2,3^, nor the big cup-shaped flowers that became fashionable in the West^11^, but it found its way to European gardens, escaped them, and became the only successfully naturalized tulip species in Europe. By the time Linnaeus described the species in 1753, it already deserved the epithet *sylvestris*, “wild”. From the 1750s onwards, *T. sylvestris* started being reported to grow wild across central and northern Europe^21,22^.

Based on the latest taxonomic treatment of the genus *Tulipa*, three subspecies are provisionally accepted under *T. sylvestris*, two of which occur in Europe: the diploid subsp. *australis* (Link) Pamp. (2x = 24) and the tetraploid subsp. *sylvestris* (2x = 48)^11,23^*.* The high number of local taxa that have been synonymized under these two subspecies (e.g., *T. celsiana* Red.*, T. biebersteiniana* Schult. & Schult.f.*, T. griesebachiana* Pant., *T. pumila* Moench and 102 more synonyms^24^;) reflect the high morphological diversity of *T. sylvestris*, which has the widest distribution within genus *Tulipa*^11^*.* Sometimes the two subspecies are treated at the species level (e.g.,^25–27^). A third subspecies, subsp. *primulina* (Baker) Maire & Weiller, also sometimes treated at the species level (e.g.,^23^), is a North African taxon and is not considered herein.

Subsp. *australis* is widely distributed, extending from the Mediterranean northwards to France and Switzerland and eastwards towards Belarus, Ukraine and Russia up to Central Asia^11^. The native distribution of subsp. *sylvestris* is uncertain, suggested to be confined to Italy, Sardinia, Sicily and NW. Libya^11^. Subsp. *sylvestris* is widely naturalized across Europe, including France, Switzerland, Austria, Germany, Poland, Belgium, the Netherlands, Denmark, Sweden, Norway and Great Britain^24,28,29^. Subsp. *australis* is a mountain plant growing in poor soils, in (semi)dry grasslands, pastures and rocky areas, whereas *sylvestris* is primarily a plant of lowland secondary habitats, growing in rich soils and areas that have been fertilized in the past, including (remnants of) historical gardens, margins of cultivated fields, vineyards, olive groves and other orchards, grassy places, and also woods, bushes, sometimes near rivers^21,25,28–34^. In several European countries *T. sylvestris* is reported as an invasive^35^, but sometimes its effects are regarded to be positive in terms of cultural and historical significance^36,37^. In the Netherlands, for example, it is an element of the so-called stinzenflora, i.e. spring flowers that were introduced as ornamentals in the past and today grow wild in historical gardens^37^. In some countries it is even designated as a species meriting conservation, for example in the Netherlands^38^, Germany^39^ and Belgium^40^.

Historical research on tulips is extensive, but has focused on those species that came from the Ottoman Empire and gave birth to modern tulip cultivars. *Tulipa sylvestris*, however, had a different introduction route, which is poorly known and perplexed because the introduced plants became widely naturalized. Here we reconstruct the European history of introduction of *T. sylvestris.* We follow the journey of the first plants that were brought from the Mediterranean to northern Europe, revealing the places, dates and botanists involved. We also discuss the species’ complex taxonomy based on our historical findings.

## Methods

Literature on tulips contains a plethora of scientific and non-scientific sources, the latter sometimes of questionable accuracy. We therefore focused our research on original sixteenth-century textual and visual evidence, including botanical publications, herbarium specimens, collections of drawings and historical archives. Tracing botanical species in pre-Linnaean texts is challenging, as a single plant name was often attributed to several botanical species, while a single species could have many different plant names. We thus started with compiling a list of “trusted” names for *Tulipa sylvestris,* which we retrieved from sixteenth-century herbarium specimens, the taxonomic identification of which we herein verified. For this we searched for specimens of *Tulipa sylvestris* in all surviving sixteenth-century herbaria^41^ for which species lists have been published and/or images of the specimens are available. Using the names of the herbarium specimens as keywords, we screened the original works of major sixteenth-century botanists, among which Clusius, Dodoens, de Lobel, Gessner and also 25 editions of Matthioli’s *Commentaries on Dioscorides* published between 1549 and 1600 in Italian, Latin, French and German. We also considered publications of the early seventeenth century by authors active during the sixteenth century, including Johann Bauhin’s *Historia Plantarum Universalis* which was published by his son-in-law almost 40 years after Bauhin’s death^42^. We further sought historical evidence of plant material exchange between southern and northern European sixteenth-century botanists in surviving archives, namely the Aldrovandian manuscripts kept at the University Library of Bologna, the digitized mail correspondence of Clusius (https://clusiuscorrespondence.huygens.knaw.nl/), and the Early modern physicians’ letters of the German speaking area (www.aerztebriefe.de). Names retrieved from textual, visual and archival sources were gradually added to the list of keywords in order to achieve maximal traceability. Regarding the Aldrovandian manuscripts, we consulted the digitized volumes at http://moro.imss.fi.it/aldrovandi/ and for the undigitized volumes, we screened the manuscript titles published by Frati et al.^43^ and requested to the University library of Bologna images of the originals for all catalogues of plants that Aldrovandi sent to northern European naturalists during the second half of the sixteenth century.

To familiarize with the morphological diversity of *Tulipa sylvestris* sensu lato across Europe, we carried out observations on ca. 400 specimens of wild and cultivated plants from around Europe, kept at Naturalis Biodiversity Center in Leiden, the Netherlands (L). Images of these specimens can be viewed at https://bioportal.naturalis.nl/, by using the advanced search and entering “Tulipa” under genus and one of the following terms under epithet: “sylvestris”, “australis”, “primulina”, “biebersteiniana”, “celsiana”.

We created a map in Adobe Illustrator 26.0.3 depicting the original and naturalized distribution of *Tulipa sylvestris* in Europe and the range of the two subspecies based on floristic sources and distribution databases of individual countries where *T. sylvestris* occurs^26,27,29,35,44–67^. Precise occurrence within a country was not always available especially in eastern and southeastern European countries.

## Results

### Early illustrations, Wieland and Gessner, 1540–1550 s

**Figure 1:**
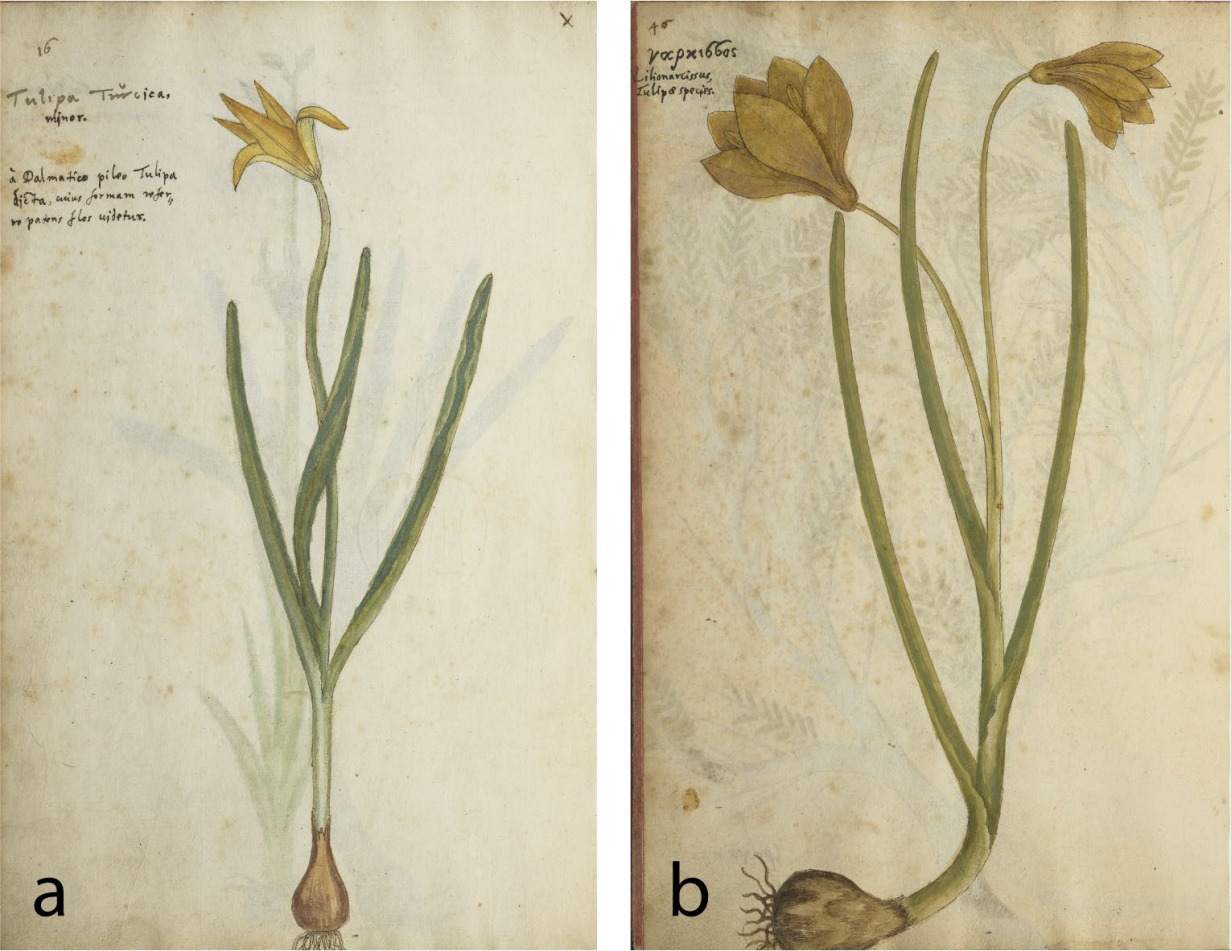
The oldest surviving illustrations of *Tulipa sylvestris* come from Italy. *Codex Kentmanus,* 1549. Klassik Stiftung Weimar, 1:16, 40:[16]v (**a**), 2:46, 172:[82]v (**b**).

The oldest surviving illustrations of *Tulipa sylvestris* are two watercolors contained in a two-volume handwritten manuscript from 1549, known as the *Codex Kentmanus* (Table 1). This manuscript was compiled by the German physician Johannes Kentmann (1518–1574), who studied medicine in Padua, Venice and Bologna between 1547 and 1549. The first watercolor is named “*Tulipa Turcica*” and depicts a slender plant bearing a single flower with five tepals (Fig. 1a). This unrealistic representation—monocots do not have five tepals—is accompanied by Kentmann’s uncertainty on the identity of this plant. In his annotations, probably compiled somewhat later (1550–1554), he wrote: “*The Turks call this plant in their barbarous tongue ‘Tulipa’; what it is I do not know*”^68,69^. The epithet “*Turcica*” implies an Ottoman origin, but all images in the first volume of the *Codex Kentmanus* were drawn after plants that Kentmann observed in Italy^69^. The paper sheet on which this “*Tulipa Turcica”* was made carries a watermark depicting a triple mountain and cross, which originates from Padua^69^ (see also^70^), where Kentmann lived and drew several plants of the city’s botanical garden^69^. During his two-year stay, Kentmann also travelled elsewhere in Italy, observing and drawing interesting and unknown plants^68,69^. The second watercolor of *T. sylvestris* in Kentmann’s manuscript is named “*νάρκισσος [nárkissos]; Lilionarcissus, Tulipae species*”, and depicts a robust double-flowered plant (Fig. 1b). It is included in the second volume of *Codex Kentmanus*, which only partly contains plants that Kentmann observed in Italy^69^. An Italian origin seems likely for this illustration as well, but it was unfortunately drawn on a sheet without watermark, nor is it accompanied by annotations. Therefore, we do not have actual evidence about its provenance.

**Table 1:** *Tulipa sylvestris* in sixteenth-century botanical books and drawing collections.

Author/compiler	Source	Date	Type of evidence	Volume & page	Original name(s)	Morphology	Reference/link
Leonhart Fuchs (1501–1506) artist: Heinrich Füllmaurer? (ca. 1500–1547/8)	Codex Fuchs	1543–7?	Image	11.122: 291	Narcissus serotinus luteus nondum dehiscens herbaceus ve Geler spater beschlossner oder griener narciss	Slender single-flowered	^79^
Leonhart Fuchs artist: possibly Albrecht Meyer (ca. 1510-after 1561)	Codex Fuchs	1543–8?	Image	11.122: 293	Narcissus serotinus luteus dehiscens; Geler spater offner narciss	Slender single-flowered	
Johannes Kentmann (1518–1574)	Codex Kentmanus	1549	Image	1: 16	Tulipa Turcica	Slender single-flowered	^68^
		1549	Image	2: 46	νάρκισσος; Lilionarcissus, Tulipae species	Robust double-flowered	
Pietro Antonio Michiel (1510–1576)	I Cinque Libri di Piante	ca. 1550–1576	Text, image	2: 21	Narcisi gialli da volgari – Tulipa spetie	Double-flowered	^80^
Conrad Gessner (1516–1565)	Historia Plantarum	ca. 1555–1565	Image (by unknown artist)	2: 466b r	Narcissi Lutei odorati ex genere Tulipanor[um?]	Three robust plants, fruiting, single- and double-flowered	http://digital.bib-bvb.de/webclient/DeliveryManager?custom_att_2=simple_viewer&pid=5331430
	De Hortis Germanis	1561	Text	213	Tulipa Turcica^a^	–	^10^
Johannes Kentmann	Kreutterbuch	1563	Image	64	Gel Wolrichent Narcissus	Four robust single-flowered plants	https://digital.slub-dresden.de/werkansicht/dlf/230814/1
			Image	78v	Tulipa turcica	Slender single-flowered	
Jacques van den Corenhuyse († after 1584) or Pieter van der Borcht (ca. 1530–1608)?	Libri Picturati core collection	1565–1567/8	Image	A30: 056v	Tulipa parva lutea. Monspell[iensis]	Three slender single-flowered plants and one fruit	^89^
Unknown	Libri Picturati	1566 the earliest	Image	A30: 062	unnamed	Two slender single-flowered plants	
Rembert Dodoens (1517–1585)	Florum	1568	Text, image	196, 198	Tulipa minor	Two slender single-flowered plants	^71^
Matthias de Lobel (1538–1616)	Stirpium Adversaria Nova	1571	Text	51	Norbonensis LilioNarcissus Luteus Montanus Tulipae species	Two slender single-flowered plants	^93^
Ulisse Aldrovandi (1522–1605)	Tavole Acquerellate	pre-1576?^b^	Image	8: 97	Lilio narcissus luteus montanus. Narcissus luteus. Narcisso lilium luteum. Tulipa lutea	Robust single-flowered	http://aldrovandi.dfc.unibo.it/pinakesweb/compdetail.asp?compid=3434
Matthias de Lobel	Plantarum seu Stirpium Historia	1576	Text, image	63	Narbonensis Lilionarcissus luteus montanus, Tulipae species minor Monspeliensis herbariorum	Two slender single-flowered plants	^97^
			Text, image	63	Bononiensis Lilionarcissus luteus, sive Tulipa	Robust double-flowered	
Matthias de Lobel	Kruydtboeck	1581	Text, image	160	Gele berg- Lelie-Narcisse van Languedoc gheheeten in Nederlandt Tulipa van Montpelliers. In Latijn Narbonensis Lilio- Narcissus luteus montanus en de Kleyne Tulipa van Dodonaeus	Two slender single-flowered plants	^96^
			Text, image	161	Gele Lelie-Narcisse van Boloignien gheheeten Tulipa van Boloignie. In Latijn Bononiensis Lilio-Narcissus luteus sive Tulipa Boloniensis	Robust double-flowered	
Andrea Cesalpino (1525–1603)	De Plantis Libri XVI	1583	Text	10: 21	Lonchitis (quidam Narcissum luteum vocant)	–	https://bibdigital.rjb.csic.es/idurl/1/13478
Carolus Clusius (1526–1609)	Rariorum aliquot stirpium	1583	Text		Tulipa Apenninea sive Bononiensis	–	^13^
Joachim Camerarius (1534–1598), Pietro Andrea Matthioli (1501–1578)	De Plantis Epitome Utilissima	1586	Text, image	958	Narcissus VIII. Lilionarcissus Bononiensis. Tulipa minor. Graecis, λειριονάρκισσος πολύκλωνος	Robust triple-flowered	^114^
	Matthioli’s Commentaries, German edition	1586	Text, image	443	Narcissus IX. Tulipa Bononiensis	Robust triple-flowered (and four-flowered mentioned in text)	^115^
Joachim Camerarius	Hortus Medicus et Philosophicus	1588	Text	125	Tulipa Narbonensis et Tulipa Bononiensis	–	^102^
Matthias de Lobel	Icones Stirpium	1591	Image	1: 124	Narbonensis Lilio-Narcissus luteus montanus, & parva Tulipa Dodonaei	Two slender single-flowered plants	https://bibdigital.rjb.csic.es/idurl/1/13266
			Image	1: 125	Bononiensis Lilio-Narcissus luteus, sive Tulipa Boloniensis	Robust double-flowered	
John Gerard (1545–1612)	Herball	1597	Text, image	116–7	Tulipa Bononiensis, Italian Tulipa, Tulipa of Bolonia	Illustration: single flowered text: robust single-, double-, triple- or more- flowered	^74^
			Text, image	116–7	Tulipa Narbonensis, French Tulipa	Robust double-flowered	
Carolus Clusius	Rariorum Plantarum Historia	1601	Text, image	150–1	Tulipa Apenninea	Two slender single-flowered plants	^14^
			Text, image	151	Tulipa Narbonensis	Robust double-flowered	
			text	151	Tulipa Hispanica	–	
Basilius Besler (1561–1629)	Hortus Eystettensis	1613	Image	–	LilioNarcissus Bononiensis. Gelb wol-riechend Tulipan	Robust triple-Flowered	–
Crispijn van den Passe (1564–1637)	Hortus Floridus	1614	Text, image	20, 91	Tulipa Bononiensis. Tulipa de Montpeliers	Robust single-flowered	https://bibdigital.rjb.csic.es/idurl/1/13646
Caspar Bauhin (1560–1624)	Pinax Theatri Botanici	1623	Text	63	Tulipa minor. I: Tulipa minor lutea Italica. an Narcissus octavus. Narcisso Constantinopolitano primo Matthioli. Lilionarcissus Bononiensis luteus, sive Tulipa Lob. Lilionarcissus Bononiensis, Eyst. & λειριοναρκισσος πολύκλονος, Cam. in Matthiolum. Lonchitis, Caes. Tulipa Apenninea Clus. hist. Italica prima, Taber. Tulipa Bononiensis, Ger. quae plerumque polyclonos, Cam. V: Tulipa minor lutea Gallica. Tulipa minor Narbonensis, Dodon. Narcissolilium luteum, Ad. Lilionarcissus Narbonensis luteus montanus, Lob. Tulipa Narbonensis, Clus. pan. hist. Cam. Tulipa Italica secunda, Tab. VI: Tulipa minor ex luteo purpurascens. Tulipa Hispanica, Clus. pan. & hist	–	^19^
Johann Bauhin (1541–1613)	Historia Plantarum Universalis	1651^c^	Text, image	2: 677	Tulipa minor lutea Narbon [ensis]	Slender single-flowered	^42^
			Text, image	2: 678	Tulipa lutea Bononiensis	Robust triple-flowered	

Morphology is given when an image (illustration) is available. The indication “robust” or “slender” is an approximate estimation of flower size and leaf width. Links to the original sources are provided where available online in the last column or the list of references.

^a^Refers to Kentmann’s illustration of 1549.

^b^Dating estimated herein based on the fact that de Lobel and the epithet “*Bononiensis*” are not mentioned in the plant names.

^c^Published posthumously.

Kentmann’s illustrations reached the scholarly circles through the Swiss botanist Conrad Gessner (1516–1565), who borrowed Kentmann’s manuscript between 1554 and 1555 ^10,69^. Besides publishing the first scientific description of a (red) tulip, Gessner^10^ also wrote about *Tulipa sylvestris* based on Kentmann’s “*Tulipa Turcica*” and acknowledged Kentmann as the source of origin for the name “*Tulipa*”. In his notes about *T. sylvestris*, Kentmann^68^ wrote that the name “*Tulipa*” is owed to the shape of the flower that refers to a Dalmatian cap (“*pileoli Dalmatici*”), an information that Gessner^10^ and other authors later reproduced, e.g.,^71–73^. Modern authors (e.g.,^1^) have argued that the name “Tulipa” derived from the Ottoman “turban” as a result of a misunderstanding, in which presumably de Busbecq was involved. But, although this information is plausible, we could not find it in sixteenth-century literature. Interestingly, towards the end of the century the English herbalist John Gerard (1545–1612) provided some insights here, writing that *“*after some days in bloom the tepal tips and margins in the flowers of *T. sylvestris* turn backwards resembling a Dalmatian or Turk’s cap, called *Tulipan, Tolepan, Turban*, or *Turfan,* from which the plant took its name”^74^ (Table 1).

**Figure 2:**
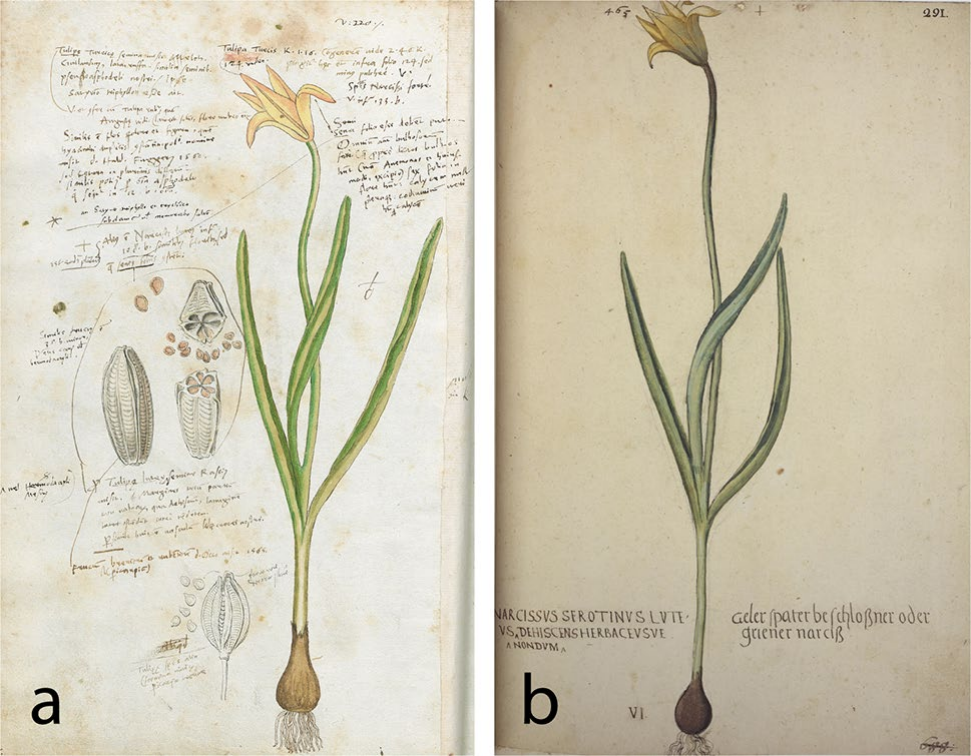
Kentmann’s illustration of *Tulipa sylvestris* was influential in sixteenth-century botany. Conrad Gessner kept a copy for his *Historia Plantarum* and drew on it the seeds he received from Melchior Wieland. University of Tartu, call nr: Mscr 55, f. 3v (**a**). An early watercolor found in the collection of Leonhart Fuchs, also presents remarkable similarity to Kentmann’s illustration. It is suggested to have been made by Heinrich Füllmaurer in ca. 1543–1547, but it is possibly of a later date. Austrian National Library, Cod. 11,122, p. 291: Historia stirpium (“Codex Fuchs”) (**b**).

Referring to Kentmann’s *“Tulipa Turcica”*, Gessner^10^ provided a rough morphological description of *T. sylvestris* and, more interestingly, mentioned that he received seeds of this plant from Guilandinus Borussus, the latinized name of Melchior Wieland (ca. 1520–1589). Wieland was a Prussian botanist who took up his studies in Königsberg in 1544/45, continued them in Italy and settled in Padua, where in 1561, he was appointed head of the city’s botanical garden, a position he held until his death^75,76^. When Gessner borrowed Kentmann’s manuscript he kept copies of 142 illustrations of plants that caught his attention, among which also *T. sylvestris*^77^ (Figs. 1a and 2a). Since Kentmann had already left Italy, Gessner asked Wieland to send him seeds of this plant from Padua. It was probably somewhere between ca. 1554 and 1559 that Wieland’s seeds arrived in Zurich. We base this on circumstantial evidence, as in 1554 Gessner saw Kentmann’s illustration and we know that he corresponded with Wieland already since at least 1556 (Gessner to Wieland, 3rd May, 1556, www.aerztebriefe.de/id/00045404), and apparently must have hosted him in Zurich even earlier since Gessner in his letters called him his former “house guest” (*hospes*)^78^. In spring 1559, at the latest, Wieland left Padua to embark on a ca. two-year field trip to the Orient, as we know that in the summer of that year he had already travelled through Istanbul to Cairo (Wieland to Aldrovandi, 2nd June, 1559, www.aerztebriefe.de/id/00016516). As far as we know, this was the first intended introduction of *T. sylvestris* northwards in Europe. Gessner must have carefully studied Kentmann’s illustration over the years, as can be seen in his personal notes on the illustration (Fig. 2a). He noted the morphological difference of this tulip from the (famous) red tulip that he had observed in Augsburg in 1559, and that this was rather more similar to another plant that he had received from Ulrich Fugger in 1560. He also drew Wieland’s seeds on the paper sheet and noted their morphological difference from another yellow tulip (“*Tulipae luteae*”) that he had received from the French surgeon and collector Nicolaus Rassius (Rassé). This might have been a yellow-flowered variety of *T. gesneriana* from Rassius’ garden in Paris.

Another important botanist of the early sixteenth century, the German Leonhart Fuchs (1501–1506), also had two illustrations of *Tulipa sylvestris* (Table 1)*,* but their dating as of 1543–1547 and 1543–1548^79^ is questionable. The plant layout in the illustration dated 1543–1547 (Fig. 2b) is remarkably similar to Kentmann’s *“Tulipa Turcica”* from 1549 (Fig. 1a), although evidently drawn by a different hand, presumably that of Fuchs’ illustrator Heinrich Füllmaurer^79^. It seems however unlikely that Fuchs, who never travelled to southern Europe, possessed already in the 1540 s a plant still unknown to northern Europeans (see also^70^ on Fuchs’ misleading identifications of Mediterranean plants). Moreover, Fuchs wrote that *T. sylvestris* was frequent in German gardens^79^, which again is inaccurate for this early period. This observation, however, might have been written later, or referred to daffodils in general.

**Figure 3:**
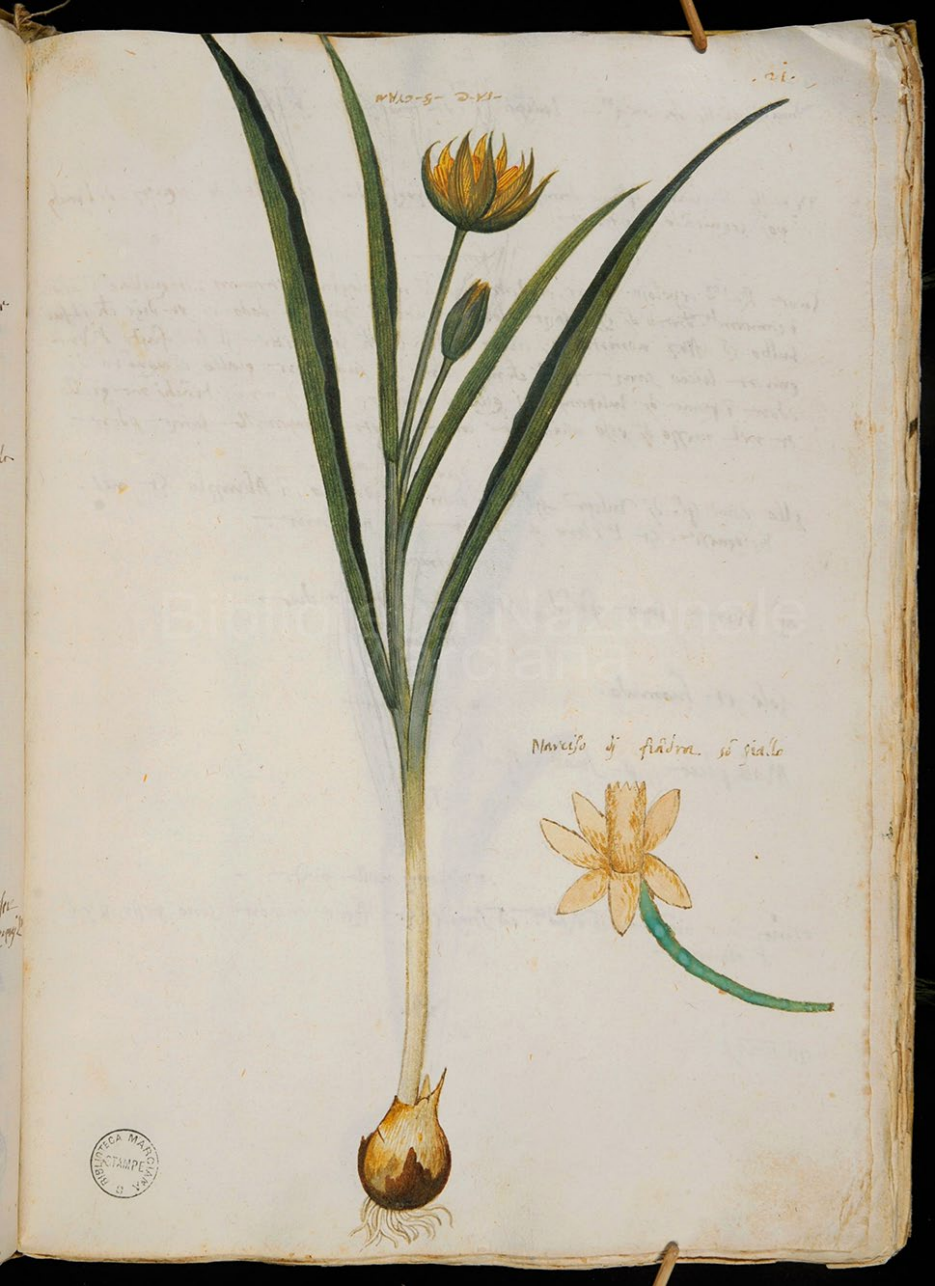
Watercolor illustration of *Tulipa sylvestris* in *I Cinque libri di piante*. This manuscript was compiled by Pietro Antonio Michiel in Venice in ca. 1550–1576. It. II, 29 (= 4863), f. 21r, Biblioteca Nazionale Marciana.

One more early illustration of *Tulipa sylvestris* survives in another handwritten manuscript of Italian origin, *I Cinque Libri di Piante,* compiled by the Venetian patrician Pietro Antonio Michiel from about 1550 to his death in 1576^80^. Michiel’s life-long masterpiece contains images of the plants that grew in his famous garden outside Venice and in the garden of Padua, which he curated between 1551 and 1555^80^. *T. sylvestris* appears in this manuscript as part of a group of yellow daffodils under the name “*Narcisi gialli da volgari—Tulipa spetie*” (“Yellow daffodils in the vernacular—Tulipa species”) (Table 1, Fig. 3). Michiel wrote that this plant grew on the Santo Angelo mountain in Abruzzo and in Bologna. A catalogue of the plants cultivated in Michiel’s garden surviving in the Aldrovandian manuscripts includes several daffodil species (“*Narcissi varie spes*”;^81^), under which *T. sylvestris* is likely also meant.

### Early herbarium specimens: Bologna, 1550 s

**Figure 4:**
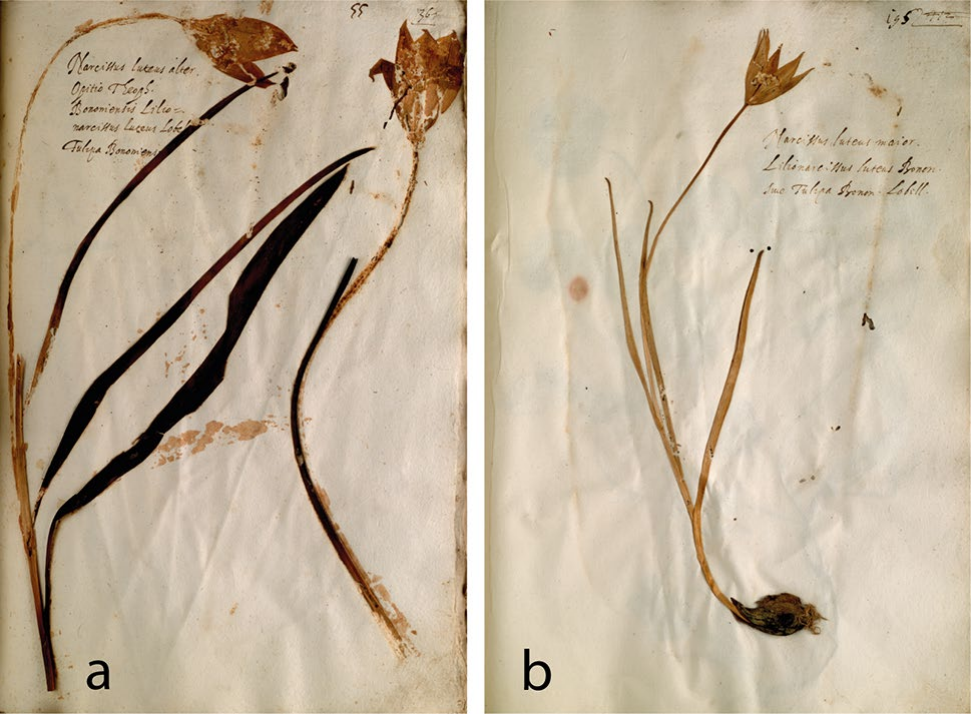
The oldest surviving specimens of *Tulipa sylvestris.* They are contained in the herbarium of Ulisse Aldrovandi (**a**): 1552, vol. IV, c. 55r, (**b**): 1553, vol. VI, c. 195r. Orto Botanico & Erbario, Universita di Bologna.

The oldest surviving specimens of *Tulipa sylvestris* also come from Italy, Bologna in specific (Table 2). Two specimens are dated 1552 and 1553 and are included in the herbarium of Ulisse Aldrovandi (1522–1605)^82,83^, one of the most prominent naturalists of the sixteenth century, appointed professor of botany in Bologna from 1561 and for the subsequent almost 40 years. The herbarium of Aldrovandi contains a total of 18 tulip specimens, some of which originated from Bologna and others from Padua. Aldrovandi probably received the latter from Wieland, as evidenced from their surviving letters, which indicate exchange of plants and tulip bulbs between the two since at least 1554. The specimens of *T. sylvestris* both came from Bologna, the 1552 specimen representing a robust double-flowered plant (Fig. 4a), and the 1553 specimen a smaller, more slender, single-flowered plant (Fig. 4b). A third early specimen from Bologna is contained in the herbarium of Francesco Petrollini (Erbario B;^83–85^), dated pre-1553^86^ (Table 2).

**Table 2:** Specimens of *Tulipa sylvestris* preserved in sixteenth-century herbaria.

Compiler/herbarium name	Volume & nr	Dating herbarium	Dating volume	Origin herbarium	Origin specimen	Original name(s)	Morphology	Subspecies	Depository
Ulisse Aldrovandi (1522–1605)	4:55	1551–1586	1552	Bologna	Bologna	Narcissus luteus alter. Opitio Theoph. Bononiensis Lilionarcissus luteus Lobelii. Tulipa Bononiensis^a^	Robust double-flowered	*sylvestris*	Orto Botanico & Erbario, Universita di Bologna, Italy. Erbario Aldrovandi vol. IV, c. 55r
	6: 195	1551–1586	1553	Bologna	Bologna	Narcissus luteus maior. Lilionarcissus luteus Bonon[iensis] sive Tulipa Bonon[ensis] Lobell[ii]^a^	Slender single-flowered	*australis*?	Orto Botanico & Erbario, Universita di Bologna, Italy. Erbario Aldrovandi vol. VI, c. 195r
Felix Platter (1536–1614)	2: 123	ca. 1552–1614	–	Basel	–	Tulipa lutea	–^b^	–^b^	Burgerbibliothek Bern, Switzerland. BBB ES 70.2 (^38^)
	2: 107	ca. 1552-1614	–	Basel	–	Tulipae	Slender single-flowered	*australis*?	Burgerbibliothek Bern, Switzerland. BBB ES 70.2 (^108^)
Francesco Petrollini Erbario B	3: 839	pre-1553	–	Bologna	–	Narcissus luteus major	robust single-flowered	*sylvestris*	Biblioteca Angelica, Rome, Italy. “Erbario Cibo”
Francesco Petrollini, En Tibi herbarium	9	ca. 1558	ca. 1558	Bologna	–	Narcissus luteus	robust double-flowered	*sylvestris*	Naturalis Biodiversity Center, Leiden, The Netherlands. L.2110826
Leonhard Rauwolf (1535?-1596)	3: 169	1560–1575	1563	northern Italy	northern Italy	Narcissus luteus	robust single-flowered	*sylvestris*	Naturalis Biodiversity Center, Leiden, The Netherlands
Andrea Cesalpino (1525–1603)	603	(1555–)1563	(1555–)1563	Pisa	–	Hemerocallis altera	Single-flowered	*sylvestris*	Museo di Storia Naturale—Collezioni Botaniche, Università di Firenze, Italy
Caspar Bauhin (1560–1624)	3: 69	1579–1624	ca. 1600–1621^c^	Basel	Montpellier & Hort de Dieu, Cevennes ^c^	Tulipa minor Gallica	Three slender single-flowered plants	*australis*	Herbarien Basel, Universität Basel, Switzerland. BAS-B03-069
Unknown compiler, Erbario A	214	–	–	–	–	–	robust single-flowered	*sylvestris*	Biblioteca Angelica, Rome, Italy. “Erbario Cibo”

The indication “robust” or “slender” is an approximate estimation of flower size and leaf width.

^a^The plant names of Aldrovandi’s specimens were written at a later date. The uniform handwriting in this multivolume herbarium suggests that all plant names were written after the compilation of the last volume, so in 1586 the earliest. The mentioning of de Lobel’s “*Lilionarcissus Bononiensis*” (published in 1576) in the two specimens supports this hypothesis.

^b^Specimen consisting only of a flower, the rest plant parts are not present.

^c^Origin and dating of specimen estimated herein based on information on the label: “minor Monspelio a D. Cherlero : major a D. Saltzman. ex horto Dei”.

**Figure 5:**
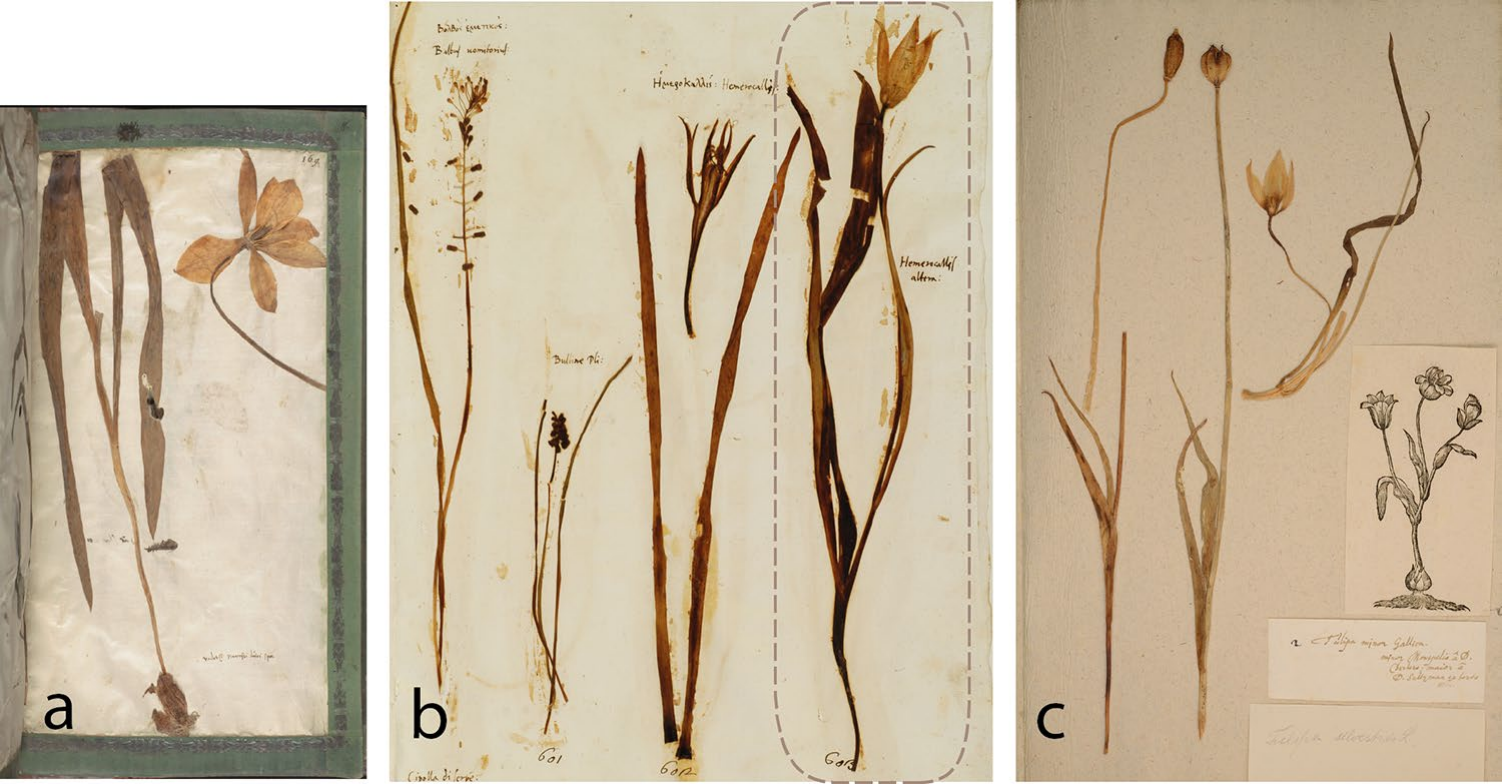
Specimens of *Tulipa sylvestris* collected by sixteenth-century botanists. Leonhard Rauwolf, northern Italy, 1563. Naturalis Biodiversity Center (**a**). Andrea Cesalpino, Pisa, 1563. Museo di Storia Naturale—Collezioni Botaniche, Università di Firenze (**b)**. Caspar Bauhin, Basel, ca. 1600–1621. Herbarien Basel, Universität Basel, BAS-B03-069 (**c**).

Six more sixteenth-century specimens of *Tulipa sylvestris* survive today, four of which are also of Italian origin. One is contained in the En Tibi herbarium, also made by Petrollini around Bologna in ca. 1558^85^ and another is part of an herbarium volume of unclear provenance (Erbario A), preserved together with Petrollini’s Erbario B (Table 2). The two combined are conventionally known as “Erbario Cibo”^84,85^. Two more specimens are dated from 1563, the first collected by the German physician and traveler Leonhard Rauwolf somewhere in northern Italy^70^ (Fig. 5a), and the latter included in the surviving herbarium of Andrea Cesalpino, made in Pisa for the bishop Alfonso Tornabuoni^87,88^ (Fig. 5b, Table 2).

Two s﻿pecimens of unknown geographical provenance are included in the herbarium of the Swiss botanist Felix Platter dated ca. 1552–1614, and a specimen of French provenance (Cevennes, Montpellier), dated most likely after 1600, is included in another Swiss herbarium, that of Caspar Bauhin from Basel (Fig. 5c, Table 2).

### The first scientific description: Dodoens and the *Libri Picturati*, 1560 s

**Figure 6:**
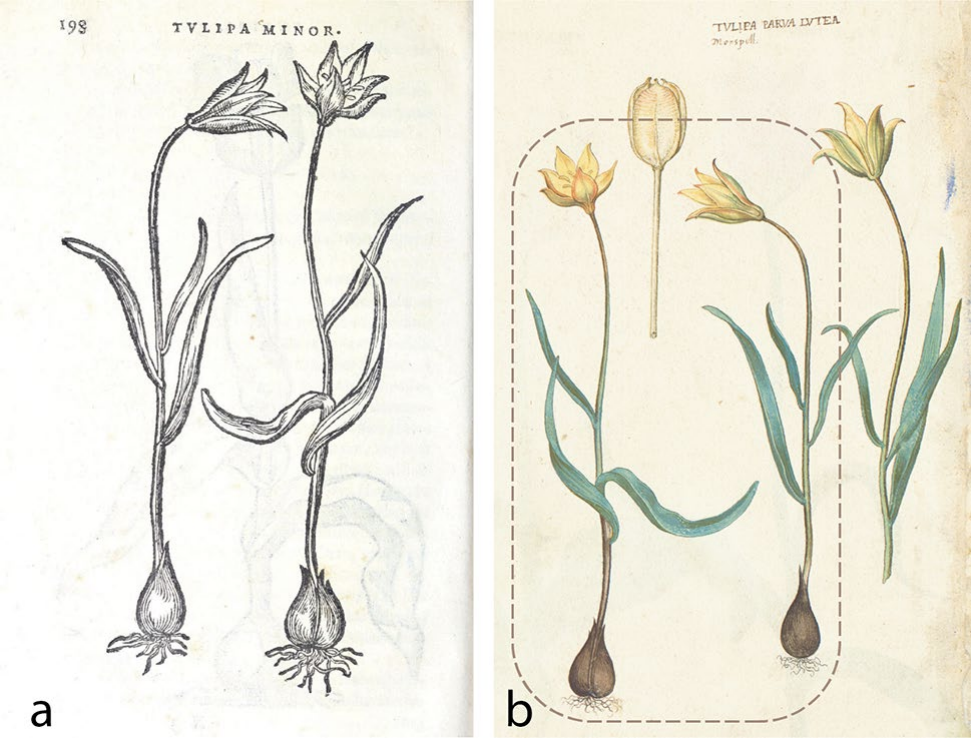
French *Tulipa sylvestris* came to northern Europe from Montpellier. Plants from this original material are probably depicted in the woodcut that accompanied the first scientific description of *T. sylvestris* published in 1568 by Rembert Dodoens. Biodiversity Heritage Library (**a**). The model used for this woodcut was a watercolor illustration from 1565-1568/9, which is part of the famous *Libri Picturati* collection. Jagiellonian Library Krakow, A30.056v (**b**).

Although Gessner^10^ had written about Kentmann’s *“Tulipa Turcica”*, it was in 1568 that *Tulipa sylvestris* was actually described for the first time in scientific literature. This was in the book *Florum* by the Flemish botanist Rembert Dodoens, latinized as Dodonaeus (1517–1585). Dodoens^71^ called *T. syvlestris* the “small tulip” (“*Tulipa minor*”) and published a short morphological description and a woodcut that depicts two slender, single-flowered plants (Fig. 6a). Dodoens wrote that these small tulips grow somewhere in southern France.

*Florum* was part of a series of popular illustrated botanical books published by the famous Antwerp-based publishing house *Officina Plantiniana* run by Christophe Plantin. The woodcut of *Tulipa sylvestris* published in *Florum* is a mirrored copy of a watercolor (Fig. 6b) contained in the *Libri Picturati*, a magnificent collection of over 1400 plant illustrations, which is Flemish in origin and presently kept in Krakow, Poland^89^. This watercolor is named “the small yellow tulip of Montpellier” (“*Tulipa parva lutea. Monspel[liensis]*”), a city located indeed in southern France, as Dodoens wrote. The watercolor is part of the core collection of *Libri Picturati* (van Uffelen and Egmond, personal communication), which is dated 1565–1568/9^90^, so it must have been shortly before 1568 that this watercolor was made. Indeed, in 1567, a year before *Florum* was published, the Mechelen-based draftsman Pieter van der Borcht (ca. 1530–1608) was commissioned by Plantin to create the images that would serve as models for the woodcuts of *Florum*, among which also the one of *T. sylvestris*^91^. Possibly van der Borcht did not paint the watercolor himself, but only transferred it to the woodblock^90^*.* Another candidate maker of this watercolor is the painter Jacques vanden Corenhuyse who made several plant illustrations for the Flemish nobleman Charles de Saint Omer or Karel van Sint Omaars (1533–1569), the original owner of the *Libri Picturati*^90^. This woodcut of *T. sylvestris* was reprinted at least 13 times in the following 80 years^91^. One more watercolor of *T. sylvestris* is found in the *Libri Picturati* and has been drawn on a paper sheet carrying a watermark from 1566 the earliest (^92^; van Uffelen, personal communication), but this image does not show stylistic similarity to Plantin’s woodcuts.

### Origin France: De Lobel—Montpellier, 1560 s

Three years after *Florum* was published, another important Flemish botanist, Matthias de Lobel or Lobelius (1538–1616), wrote that he dug out bulbs of *Tulipa sylvestris* from the Cevennes mountains (“*Dei Paradisii Iugis vocatis Sevenae Norbonensis”*), north of Montpellier, and sent them to Belgium^93^. De Lobel apparently referred to the Hort de Dieu, a valley located at the southern slope of Mt. Aigoual at the southern Cevennes, which was a botanical destination in the sixteenth and later centuries^94^. De Lobel used the name “*Lilionarcissus Norbonensis*” (Table 1), the epithet “*Norbonensis*” indicating Narbone as the geographical provenance of the plant, which corresponds to Provence and Languedoc, or generally southern France. “*Lilionarcissus*” was a name used for tulips in scholarly circles at that time^14^, but was superseded by the vernacular “*Tulipa*” that was finally established nomenclaturally by Linnaeus^20^. De Lobel probably dug out those bulbs between 1565 and 1567/8, as this period he was living in Montpellier for his medical studies and eagerly botanized the city’s surroundings^95^.

De Lobel mentioned that he sent the bulbs to friends in Antwerp, but who were they? Perhaps the most well-known naturalist of Antwerp was the apothecary Pieter van Coudenbergh, but unfortunately, the inventory of his famous garden^10^ was published at least five years before de Lobel sent the bulbs. It is therefore not surprising that no plant in this catalogue could be matched to *Tulipa sylvestris*, the closest record being a reference to three daffodil species (“*Narcissi tres species*”;^10^). Moreover, van Coudenbergh was not listed by de Lobel^96^ among his close acquaintances, making it rather unlikely that he was the one who received the bulbs. More interestingly, de Lobel^96^ mentioned in his Cruydtboeck two scribes from Antwerp who supported his work with their gardens. The first was Willem Martini, city scribe of Antwerp since 1565, known also for his involvement in the Dutch resistance against the Spaniards. The second was Jan van Hoboken, scribe of Antwerp at a later time, 1578–1590. It may be that any or both of these two men received de Lobel’s bulbs, but further information about their botanical activity is not known.

Besides the two Antwerp scribes, de Lobel gave a long list of plant “facilitators” in which a certain Mr. Reynoutre stands out. This name is an alias of Saint Omer, first owner of the *Libri Picturati*, where the model watercolor of the *Florum* woodcut of *Tulipa sylvestris* is included. Saint Omer was a wealthy nobleman and owner of an estate and castle in Moerkerke, near Bruges in Belgium^90^. *Florum*’s watercolor, which was later reproduced also in de Lobel’s (and Clusius’) books published by Plantin, was made in the same period (between ca. 1565 and 1568) that de Lobel dug out the bulbs in Montpellier, and this provenance is also mentioned in the watercolor’s name (“*Monspelliensis*”). It may thus be assumed that de Lobel was also the direct or indirect source of the *T. sylvestris* bulbs that Saint Omer cultivated in his garden, the material based on which the species was for the first time described by Dodoens in 1568.

### Origin Italy: Aldrovandi—Bologna and Apennines, 1570 s

**Figure 7:**
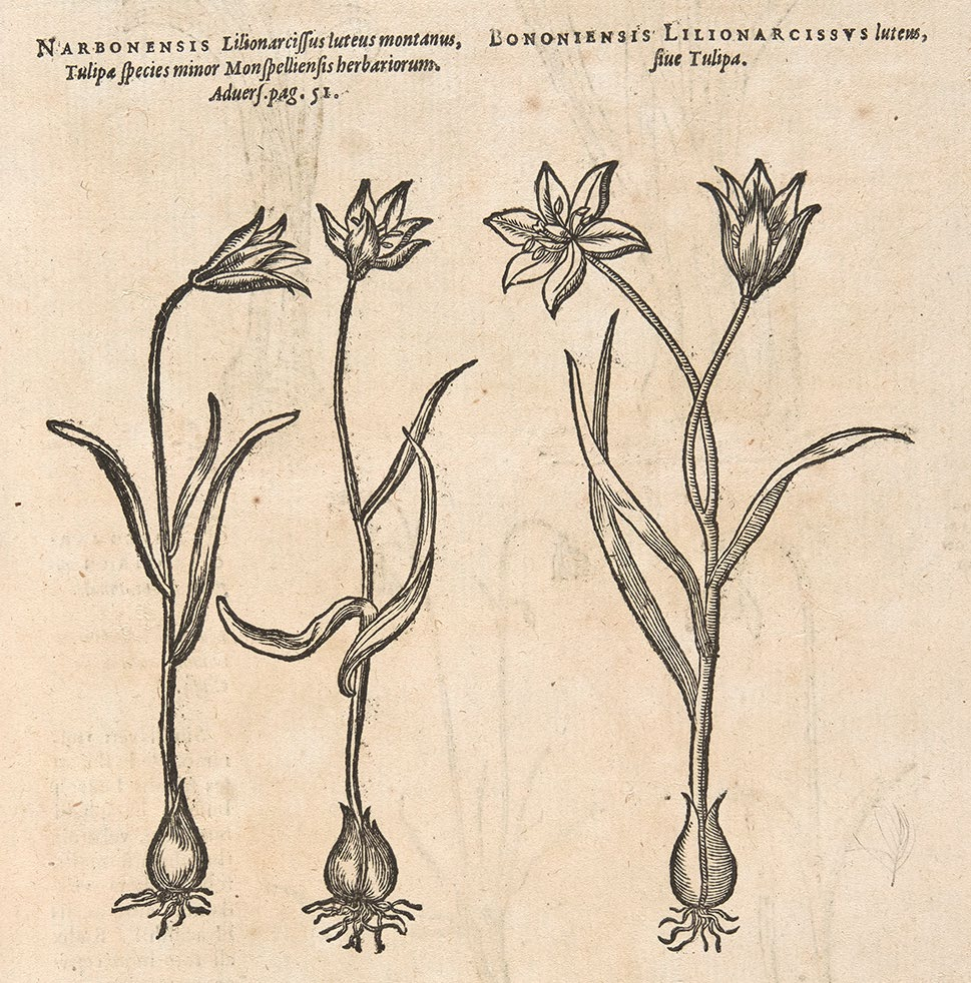
The Narbone (left) and Bologna (right) tulip. These woodcuts were reproduced many times in Plantin’s botanical publications. Here in *Plantarum seu stirpium historia* by Matthias de Lobel from 1576 (Special Collections, Wageningen University & Research – Library).

Five years after de Lobel wrote about the Narbone tulip, he introduced in literature the Bologna tulip, “*Bononiensis Lilionarcissus luteus, sive Tulipa*”^97^ (Fig. 7). He wrote that the Bologna tulip looks like the Narbone tulip “*in leaf, stem and flower, but it is fragrant, significantly more vigorous and bigger*”, the latter a detail of taxonomic importance as will be discussed below. He also added that the Bologna tulip was sometimes double-flowered, as can be seen in herbarium specimens and illustrations of the time (Figs. 1b, 4a and 7). This time, de Lobel published also two woodcuts: one for the Narbone tulip, the same as Dodoens’ woodcut from *Florum,* and one for the Bologna tulip, representing a double-flowered individual (Fig. 7). Unlike the woodcut for the Narbone tulip*,* the model used for the woodcut of the Bologna tulip could not be traced among the *Tulipa sylvestris* illustrations and specimens that survive today. Its stylistic resemblance to *Florum*’s woodcut points to another, perhaps lost watercolor that has not survived in the *Libri Picturati* collection presently kept in Krakow.

We do not know where de Lobel got his Bologna tulips from. They possibly grew since early in the city’s gardens, as Johann Bauhin (1541–1614) reported having seen them in San Salvatore^42^, one of the oldest gardens in Bologna, existing at least since the early 1550 s^98^. Aldrovandi and Petrollini already possessed specimens (Table 2) before or shortly after their graduation in the early 1550 s^98^, so it must have been in the study material of medical students at the university of Bologna. Nevertheless, this tulip is not easy to trace in the 1568–1582 inventory of the Bologna public garden, which Aldrovandi founded in 1568. Several yellow tulips are listed in this inventory, but they are all mentioned to have a Turkish origin (Aldrovandian manuscripts Ms. 002).

What we do know is that Clusius received *Tulipa sylvestris* directly from Aldrovandi, as Clusius personally recalled: “*It grows in abundance in the Apennines from where Ulisse Aldrovandi, the Bolognese professor, dug it out and sent it to me from Bologna, many years ago*”^14^. Clusius discarded de Lobel’s “*Lilionarcissus*” in favor of “*Tulipa*” and used again the same woodcuts as Dodoens and de Lobel for the French and Italian plants^13,14^. Clusius also suggested a new place of origin for *T. sylvestris* in Italy: the Apennine mountains. He first used the epithet “*Apenninea*” complementary to “Bononiensis” (“*Tulipa Apenninea sive Bononiensis*”;^13^) and later kept only the Apennine provenance, “*Tulipa Apenninea*”;^14^) (Table 1), though still mentioned that he received the plant from Bologna.

In our attempt to trace when Clusius could have received the Apennine tulip from Aldrovandi, we found a catalogue dated ca. 1574–1575^99^, containing a list of plants that Aldrovandi sent to Clusius for the imperial garden in Vienna (Aldrovandian Manuscripts, Ms. 136/05, cc. 371–374). A certain “*Tulipanum luteum*” (yellow tulip) is listed therein, which may refer to *Tulipa sylvestris* and could be the plant that Clusius remembered having received from Aldrovandi. However, the possibility of a garden tulip variety with yellow flowers (*T. gesneriana*) cannot be excluded. What is certain is that in 1571, Clusius had received seeds of both Bologna and Montpellier tulips from one of his patrons, Jean de Brancion, a rich man from Mechelen, as Brancion himself declared in his letter to Clusius on August, 3rd that year (Table 3)^100^. Clusius further dispersed this material in his network. For example, in 1577 he sent bulbs of both tulips to his German friend Joachim Camerarius, instructing him how to care for these “*Tulipas Bononieses*” and “*Mompelianas*”: *“Do not mix them together with other tulips. Because they produce lateral bulbs and spread. It is better to put them in a distinct space […] so that they don’t spread too far. Otherwise they would occupy the whole garden in a few years”*^101^. Clusius^13,14^ repeated this observation from his own garden, that these tulips produce lateral bulbs through stolons (*“tenuibus nervis in latera”,* “lateral nerves at the sides”) and extensively spread. We do not know whether the Bologna and Montpellier tulips that Camerarius received from Clusius thrived, but eleven years later both tulips were still growing in his garden in Nurnberg^102^ despite the harsh winters. Not all plant exchanges were successful though. The Dutch apothecary Willem Jasperse Parduyn (ca. 1550–1603) informed Clusius in 1596 about the rotten state of most of the plants he had received, among which also the “*Tulipa Bononiensis*” (Table 3)^103^. The Bologna tulip was not only cultivated in northern European gardens but also in Italian ones. In 1606, the prefect of the Pisa garden, Franscesco Malocchi, sent to Clusius a list of the garden’s most beautiful plants, among which the “*Tulipa Bononiensis lutea odorata*” (“scented yellow Bologna tulip”) (Table 3)^104^. Johann Bauhin reported having received the Bologna tulips in Montbeliard, N. France, from Guilhelmus Landgravius (probably William IV, Landgrave of Hesse-Kassel) and, like Clusius, he also reported the tendency of these tulips to multiply and spread^42^. The observations of these two rigorous botanists about the vegetative reproduction of *T. sylvestris* form the earliest evidence of naturalization for this species*,* a garden escape established today in the wild in many European countries.

**Table 3:** *Tulipa sylvestris* in mail correspondence of sixteenth century botanists and scholars.

Sender	Recipient	Date	Original name(s)	Reference/link
Jean de Brancion	Carolus Clusius (1526–1609)	3.8.1571	Tulipa de Boulogne et Mompelier	^100^
Carolus Clusius	Joachim Camerarius (1534–1598)	30.7.1577	Tulipas Bononienses et Mompellianas	^101^
Willem Jasperse Parduyn (1550–1603)	Carolus Clusius	1.11.1596	Tulipa Bononiensis diversa ab Hispanica et Narbonensi	^103^
Jean Sr. Robin	Carolus Clusius	10.4.1599	Tulipan de boulogne	https://clusiuscorrespondence.huygens.knaw.nl/edition/entry/1445/transcription
Francesco Malocchi (†1613)	Carolus Clusius	8.11.1606	Tulipa Bononiensis lutea odorata	^104^

### Origin Spain: Aranjuez, 1580 s?

Together with the Apennine and Narbone tulip, Clusius^14^ grouped also another plant which grew on a hill close to Aranjuez, in central Spain. This “*Tulipa Hispanica*” was “*similar to the Narbone tulip but slightly smaller”.* Clusius had not observed this plant during his Spanish travels, nor had he seen its flower, but got the information that the flowers are dark red outside. He recalled that this plant was introduced to Belgium by the gardener Francisco de Hollebeque, a distiller from Mechelen, who became gardener at the royal garden of King Philip II of Spain in Aranjuez in 1580^105^. So it was probably after that year that these Spanish tulips were brought to Belgium. Clusius^14^ noted the difficulty to cultivate this tulip, whose bulbs only gave a single leaf and gradually perished in the Belgian environment.

### History versus taxonomy

**Figure 8:**
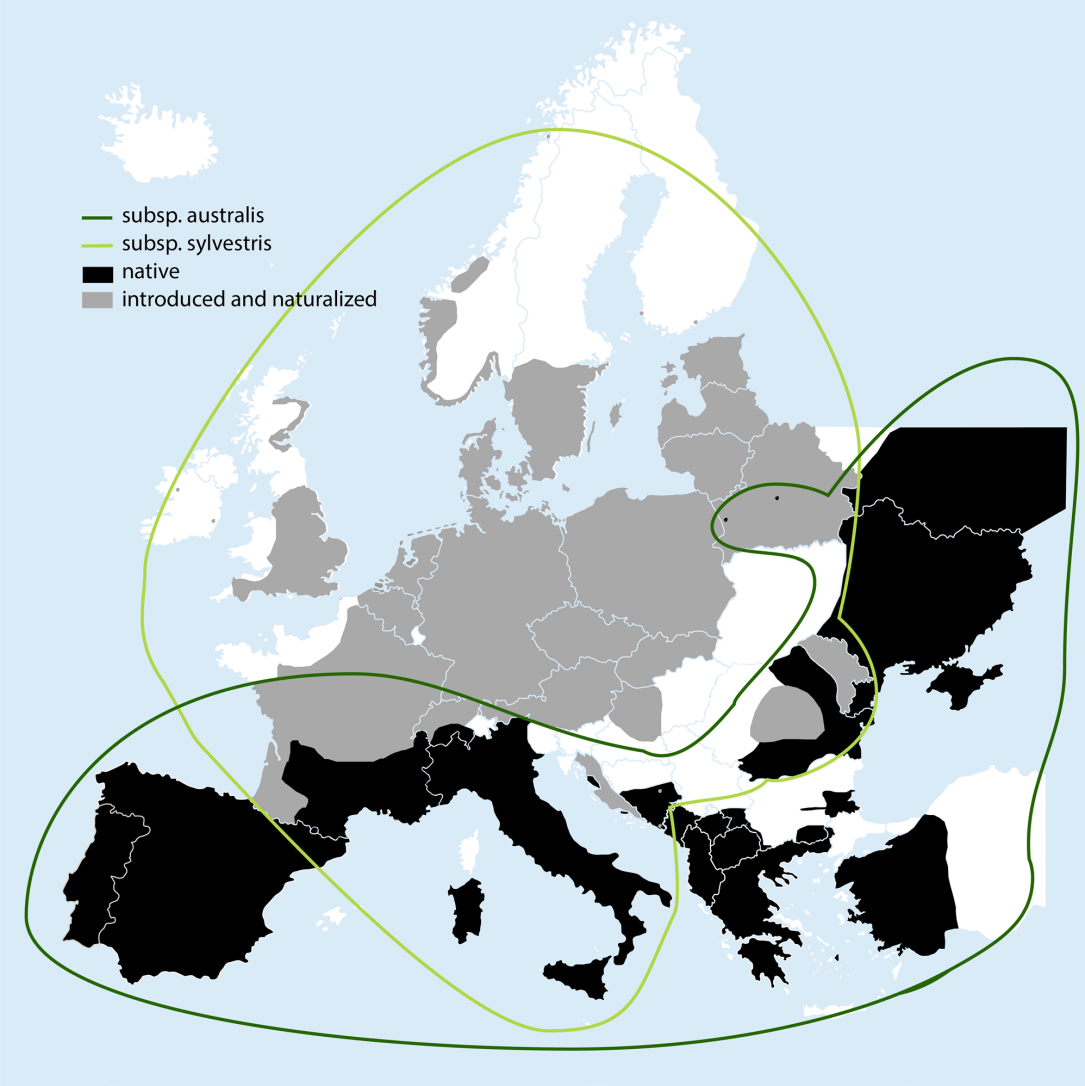
Distribution of *Tulipa sylvestris* in Europe. Black color indicates native occurrence and grey naturalized . Precise occurrence within each country is shown where available. Created with Adobe Illustrator 26.0.3, background map: Shutterstock, https://www.shutterstock.com .

**Figure 9:**
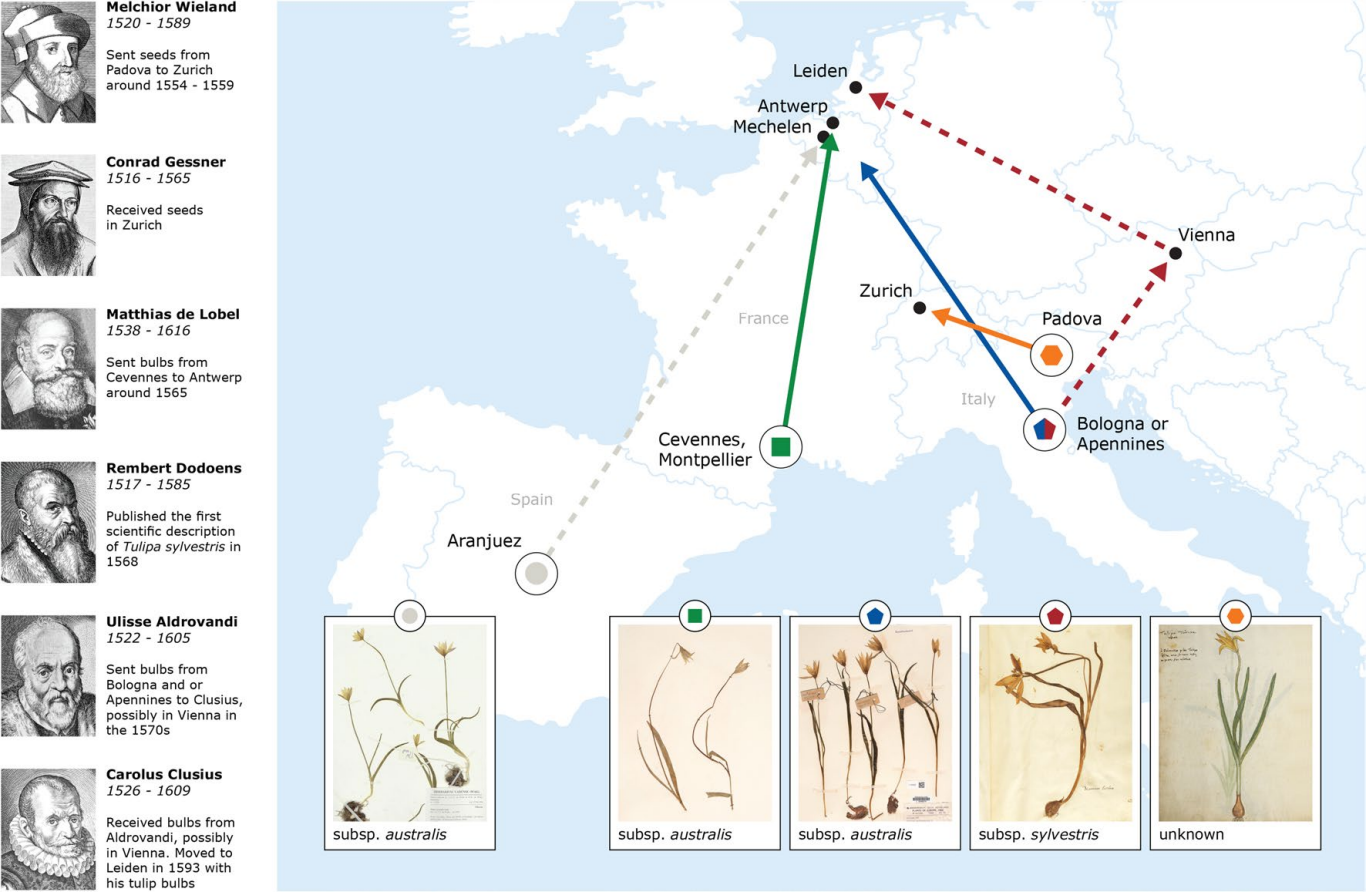
Illustrated history of introduction of *Tulipa sylvestris* in sixteenth-century Europe*.* Unlike the progenitors of modern tulip cultivars that came from the Ottoman empire (present day Turkey), the “wild tulip” (*T. sylvestris*) came from Italy and France, following the routes shown in this map. Plants from Spain were also introduced but apparently unsuccessfully (light grey). The depicted specimens (Naturalis Biodiversity Center), reflect the morphological diversity of the introduced plants: the French and Italian specimens are plants that have been collected in the Cevennes (L.1458540), Bologna (L.2110826) and the Apennines (L.1458536). The Spanish specimen (WAG.1057471) shows the presumed morphology of the plants introduced from Aranjuez. Infographic created by Alfred Heikamp with Adobe InDesign 2022 17.0 and Adobe Illustrator 2022 26.0.3. Background map: Shutterstock, https://www.shutterstock.com.

Connecting our historical findings with the current taxonomy and distribution of *Tulipa sylvestris*, we get some interesting insights. Based on the species’ current subspecific circumscription, the plants that are naturalized over Europe belong to the tetraploid subsp. *sylvestris*, while the plants that are native in the Mediterranean region belong to the diploid subsp. *australis*^11^. Subsp. *sylvestris* is apparently native also in (parts of) Italy^11^ (Fig. 8). Our findings, however, show that actually both subspecies have been introduced to the north. From the overview of historical literature, we obtained several places of origin for *T. sylvestris*: the Cevennes mountains in southern France, Bologna and the Apennine mountains in northern Italy, and Aranjuez in central Spain (Fig. 9). Looking into the taxa that grow today in these regions, we see that only the plants that grow around Bologna belong to subsp. *sylvestris*. The tulips that grow in the Cevennes, Apennines and Aranjuez belong to subsp. *australis*^21,30,46,47,106,107^ ﻿(Fig. 9). If any bulbs of *T. sylvestris* did come to Italy from the Ottoman empire, then these would have likely also been subsp. *australis*^44^ (Fig. 8).

## Discussion

Linnaeus^20^ described *Tulipa sylvestris* as the small yellow tulip of Italy and France (“*Tulipa minor lutea Italica*” and “*Tulipa minor lutea Gallica*”). He copied this information from Bauhin’s *Pinax*^19^, a monumental work that summarized plant names of sixteenth-century literature (Table 1). Our screening of original sixteenth-century sources revealed where exactly in Italy and France the first bulbs of *T. sylvestris* came from, when, and which botanists were involved in their introduction to northern Europe. The first material came from Italy in a still early period for botany as a field study^108^; Between ca. 1554 and 1559, Wieland sent seeds from Padua to Zurich to Gessner. The latter was at that time the most famous naturalist beyond the Alps, and by sending him tulip seeds Wieland possibly hoped to increase his status in the botanical scene (which he achieved shortly after, in 1561, when he became the prefect of the Padua garden). Wieland may have received his material from Bologna, from his friend Aldrovandi, who already had access to this plant in the early 1550 s. We do not know, however, if Wieland’s seeds ever grew into flowering plants in Gessner’s garden or if any derived plant material was further dispersed by Gessner to fellow naturalists. Growing tulips from seed to flower takes years, and in 1561 Gessner reported only the receipt of the seeds. And four years later, in 1565, he died. Around that year, de Lobel “opened” the second introduction path, from France, and was acknowledged by Clusius^14^, the most influential man in tulip history, as the first who introduced *T. sylvestris* to northern Europe. De Lobel had discovered subsp. *australis* in the Cevennes mountains and via Saint Omer or another route this material may have reached the creator of the *Libri Picturati* and also Dodoens, who published the first scientific description of *T. sylvestris* in 1568. In 1576, de Lobel coined the name “Bologna tulip”, but the source of his Bologna tulips remains unclear. Possibly it was again Aldrovandi, either directly or indirectly through their common network. Throughout the second half of the sixteenth century, Aldrovandi sent material of numerous plant species to his northern European correspondents. A plethora of plant catalogues kept in his surviving manuscripts in Bologna are evident of these exchanges^43^. De Lobel had lived in Bologna where he had met Aldrovandi and acquired from him both plants and botanical knowledge^96^. It is likely that during his stay in Bologna, de Lobel received information about *T. sylvestris*, however, he did not directly report this in his books, nor is any plant exchange between the two men found in the surviving Aldrovandian manuscripts. Among the plant suppliers that de Lobel^96^ mentioned in the introduction of his *Kruydtboek*, we also find de Brancion, who vigorously exchanged plants with Aldrovandi between ca. 1563 and 1568^109^. Based on the surviving Aldrovandian manuscripts, de Brancion was the only Flemish (besides Clusius) with whom Aldrovandi exchanged plant material. De Brancion was a close friend of Clusius^90^, to whom he had sent *T. sylvestris*^100^ and also of Dodoens^110^. Within this network, de Brancion could have received the Bologna tulips from Aldrovandi and sent them to de Lobel, but no actual evidence was found that supports this hypothesis. Clusius^14^ reported having received *T. sylvestris* from Aldrovandi from Bologna, from material originating from the Apennine mountains, an event that possibly happened in the 1570 s. Plants with more reddish-tinted flowers were introduced from Spain^14^, but apparently did not flourish in northern European gardens. Totally different introduction paths are also possible. For instance, it has been suggested that *Tulipa sylvestris*, being a weed of vineyards, was unintentionally introduced northwards in Europe through the grapevine export, perhaps already since Roman times^1^. This path could have acted complementarily to the intended introduction of sixteenth-century naturalists. It is even possible that also bulbs of *T. sylvestris* were among the numerous kinds of tulips that arrived from the Ottoman empire to Italian gardens in the early 1550 s, as Kentmann’s “*Tulipa Turcica*” and the 1568–1582 Bologna garden inventory imply.

Whatever the routes of introduction of *Tulipa sylvestris* may have been, it was the strong sixteenth-century Flemish botanical network, supported by Clusius, de Lobel, Dodoens, Plantin and their rich friends with gardens, such as Saint Omer and de Brancion, which facilitated the introduction of *T. sylvestris* and its spread in Europe. This network was supplied with Mediterranean plant material from two main centers, southern France and northern Italy, which were popular botanical destinations for northern European naturalists^108^. Aldrovandi from Bologna was a direct source of such material^43^. Clusius had a large network of correspondents in Europe to whom he sent tulip bulbs^15^, so we may expect that he distributed bulbs of *T. sylvestris* to more correspondents than those reported herein*.* Archives and letters between sixteenth-century naturalists contain plenty of references to exchanges of bulbs, tulips and daffodils, under which *T. sylvestris* may also be meant. Unfortunately, in most cases these vague plant names could not be linked to botanical species. It was with de Lobel’s works^93,97^ that it became evident that a group of tulips, in fact *T. sylvestris*, grew wild on European (Mediterranean) ground. De Lobel’s epithets “*Bononiensis*” and “*Narbonensis*” and Clusius’ “*Hispanica”* clearly distinguished *T. sylvestris* from the exotic garden tulips that were imported from the Ottoman empire. The Bologna origin persisted in literature and almost a century after, *T. sylvestris* is explicitly indicated as the Bologna tulip in the 1657 inventory of the Bologna public garden (“*Tulipa flore luteo minor Bononiensis”*, “the small yellow-flowered tulip of Bologna”)*,* unambiguously distinguished from the several garden tulips of various colors and shapes (“*Tulipanorum variae species, & variorum colorum*”)^111^. On the other hand, the evidence that has reached our days is dominated by the large archives of Clusius and Aldrovandi. If more information had survived about Wieland, Dodoens, de Lobel or other naturalists, we may have had another view of the introduction history of *T. sylvestris*.

The sixteenth century was the time when the study of plants shifted from a focus on medicinal uses to an interest on plant diversity and taxonomy^85,112,113^. *Tulipa sylvestris,* and tulips in general, do not possess medicinal properties and were probably of no interest to classical authors and their successors. It is therefore no wonder that Pietro Andrea Matthioli, the most published botanical author in the sixteenth-century and faithful follower of Dioscorides, wrote nothing about tulips. The interest on plants as direct object of study was gradually shaped in the course of the century. It would be no exaggeration to say that this reformation took place in parallel with the introduction of tulips in Europe; these flowers of unprecedented beauty and no apparent use, other than ornamental. Matthioli did publish woodcuts of tulips, though, some of which imaginary, in his chapter about daffodils. Only after Matthioli’s death, his opponents dared to publish their amended versions of Matthioli’s *Commentaries*^112^. In such an occasion, Camerarius^114,115^ suggested that a multi-branched daffodil of Matthioli may have actually been *T. sylvestris*, and he published a woodcut of the species representing a triple-flowered plant (Table 1).

Interestingly, de Lobel^97^ had noticed that the Bologna tulip is bigger than the French one. One of Clusius’ correspondents, Parduyn^103^, also noted the distinctiveness of the Bologna tulip (subsp. *sylvestris*) from the French and Spanish one (both subsp. *australis*): “*Tulipa Bononiensis diversa ab Hispanica et Narbonensi*” (“the Bologna tulip, which is different from the Spanish and Narbone tulip”) (Table 3). Indeed subsp. *sylvestris* has been suggested to be an autotetraploid derived from the diploid subsp. *australis* (^11,30^, see also^106^), and as such it is expected to be larger in size. On the other hand, subsp. *sylvestris* grows in richer soils than subsp. *australis*, another factor that favors a larger plant size. Female sterility was also suggested to affect plant size in subsp. *sylvestris*^116^. De Lobel also noticed that the Bologna tulip was fragrant and more vigorous (“*vegetatiora*”) than the French one, which may imply that the former plant can easier establish in (and be favored for) a garden. He further wrote that Bologna tulips were sometimes double-flowered, a character which may also be connected to their tetraploid nature. Sixteenth-century plants from Italy were mostly visualized by double-flowered individuals (Fig. 1b, 4a and 7), sometimes even triple (Table 1). Nevertheless, populations of subsp. *sylvestris* have mostly single-flowered plants, the division of the stem being rather a random, infrequent event. Apparently, the multiple flowers, like the pleasant scent, were a desired character for ornamental purposes. In fact, subsp. *sylvestris* may have been a naturalized cultivated plant also in Bologna, as it occurs in the city environs in secondary habitats, and its native occurrence has been questioned^21,106^. Already since the sixteenth and seventeenth century it was reported being cultivated in the gardens of Bologna and other Italian cities^42,104,111^. It could even be that the tetraploid Bologna tulip derived from material that was brought to the city’s gardens from the Ottoman empire in the 1540–1550 s. The two subspecies have distinct ecological and altitudinal preferences and do not co-occur in the wild. The different forms illustrated in sixteenth-century literature, slender vs. robust, single- vs. multi-flowered plants (Tables 1 and 2, Figs. 1, 4, 5 and 7), may correspond to these two subspecies, the diploid *australis* and the tetraploid *sylvestris,* but this is not always straightforward. Since both subspecies were brought together in sixteenth-century gardens, we would expect that triploids also occur around Europe, which is supported in literature^33,117^.

The Apennine origin of Clusius^14^ is somewhat enigmatic. Clusius lumped together the Apennine and Bologna tulips (Table 1)^12–14^, but the first should belong to subsp. *australis* and the latter to subsp. *sylvestris*. One could assume that the Apennine origin was a misunderstanding of Clusius and that Italian plants came only from Bologna. Indeed, Clusius^14^ wrote that the Narbone tulip looked like the Apennine tulip, but it was “generally smaller”, a statement that seems confusing considering that the *australis* plants of the Cevennes would not be smaller than the (also *australis*) plants of the Apennines, but they would be smaller than the *sylvestris* Bologna plants. In physically examining the two specimens preserved in Aldrovandi’s herbarium in Bologna, we noticed that only one of them (Fig. 4a) can be identified with certainty as subsp. *sylvestris*. The other specimen (Fig. 4b) can rather be identified as subsp. *australis*^25,32,33^, but depending on the identification source used it can also fall within the range of both *australis* and *sylvestris*^29^ or even be *sylvestris*^118^. Clusius^14^ also wrote that the flowers of the Narbone tulips were greener outside compared to the Apennine tulips. This statement adds to the confusion, considering that the flowers of the plants that grow (in abundance) in the Cevennes are reddish outside, and not greenish (^29^, F. Hopkins personal communication). Plants with greenish outer tepals that belong to subsp. *sylvestris* are said to grow in the area, but are hard to find (F. Hopkins, personal communication). The *Libri Picturati* watercolor, which is probably a plant from the Cevennes, rather has greenish-red tepals (Fig. 6b). Actually, among sixteenth-century representatives of *Tulipa sylvestris*, only Clusius’ “*Tulipa Hispanica*” was mentioned to have reddish color in the outer tepals^14^, which is a main discriminant character for subsp. *australis* vs. *sylvestris* in modern floras, including the Spanish one^47^.

The above make evident that the currently accepted distinction of subsp. *sylvestris* as a naturalized tetraploid in (northern) Europe and subsp. *australis* as a native diploid in the Mediterranean (up to Central Asia) does not conform with the history of introduction of *Tulipa sylvestris,* because both diploid and tetraploid plants have been introduced northwards to Europe. The introduced plants came from three or four different locations in Italy, France and Spain and are morphologically variable, although probably Spanish plants did not flourish after their introduction (Fig. 9). A new taxonomic assessment of *Tulipa sylvestris* is needed. This will be the next step of our research, employing genomics, morphometry and ploidy.

We suggest that a combination of botanical and historical research is necessary in order to understand the complex origin and taxonomic status of naturalized plants that have a past of introduction. Recent studies point in the same direction^119–122^. Large-scale initiatives of the last decades to digitize historical archives, books and herbaria, and make them publicly available online pave the way for such interdisciplinary studies^85,121,123^. Establishing global open-access databases with more historic material can provide new tools for research in botanical history^122^. Physical access to the original material remains essential for observing details, as conducted herein for example to verify the taxonomic identification of Aldrovandi’s ca. 470-year-old herbarium specimens. Nevertheless, our focus in botanical history studies should be (remote) research on digitized images, as this enormously facilitates the study and accessibility of valuable treasures of our botanical heritage, without the risk of damaging them.

## Data Availability

Most of the data used in this article are included within the manuscript. All sixteenth-century books consulted are available online and links to their digitized content are provided in the list of references. Most of the illustrations and herbarium specimens used are available online at the websites of the libraries where they are kept. In order to get access to illustrations and specimens that are not available online, we requested copies to the libraries that hold the copyright; data availability in this case depends on the copyright holder policy.
